# The Association Between Plasma Osmolarity and In-hospital Mortality in Cardiac Intensive Care Unit Patients

**DOI:** 10.3389/fcvm.2021.692764

**Published:** 2021-07-02

**Authors:** Guangyao Zhai, Jianlong Wang, Yuyang Liu, Yujie Zhou

**Affiliations:** Beijing Anzhen Hospital, Capital Medical University, Beijing, China

**Keywords:** cardiac care intensive unit, cardiovascular, in-hospital mortality, “U”-shaped, plasma osmolarity

## Abstract

**Objectives:** Plasma osmolarity is a common marker used for evaluating the balance of fluid and electrolyte in clinical practice, and it has been proven to be related to prognosis of many diseases. The purpose of this study was to identify the association between plasma osmolarity and in-hospital mortality in cardiac intensive care unit (CICU) patients.

**Method:** All of the patients were divided into seven groups stratified by plasma osmolarity, and the group with 290–300 mmol/L osmolarity was used as a reference group. Primary outcome was in-hospital mortality. The local weighted regression (Lowess) smoothing curve was drawn to determine the “U”-shaped relationship between plasma osmolarity and in-hospital mortality. Binary logistic regression analysis was performed to determine the effect of plasma osmolarity on the risk of in-hospital mortality.

**Result:** Overall, 7,060 CICU patients were enrolled. A “U”-shaped relationship between plasma osmolarity and in-hospital mortality was observed using the Lowess smoothing curve. The lowest in-hospital mortality (7.2%) was observed in the reference group. whereas hyposmolarity (<280 mmol/L vs. 290–300 mmol/L: 13.0 vs. 7.2%) and hyperosmolarity (≥330 mmol/L vs. 290–300 mmol/L: 31.6 vs. 7.2%) had higher in-hospital mortality. After adjusting for possible confounding variables with binary logistic regression analysis, both hyposmolarity (<280 mmol/L vs. 290–300 mmol/L: OR, 95% CI: 1.76, 1.08–2.85, *P* = 0.023) and hyperosmolarity (≥330 mmol/L vs. 290–300 mmol/L: OR, 95% CI: 1.65, 1.08–2.52, *P* = 0.021) were independently associated with an increased risk of in-hospital mortality. Moreover, lengths of CICU and hospital stays were prolonged in patients with hyposmolarity or hyperosmolarity.

**Conclusion:** A “U”-shaped relationship between plasma osmolarity and in-hospital mortality was observed. Both hyposmolarity and hyperosmolarity were independently associated with the increased risk of in-hospital mortality.

## Introduction

Although the prognosis of cardiovascular diseases has greatly improved due to technological advances and innovative drug use, cardiovascular diseases still remain the leading cause of death and disability worldwide (1). Much research is still needed in the field of cardiovascular diseases, especially for severe cardiovascular diseases with high mortality (2). Cardiac intensive care unit (CICU) has been established to manage severe cardiovascular diseases, and patients admitted to the CICU are usually at great risk of adverse outcomes (3). For CICU patients, readily available risk factors are always welcomed by clinicians, which will help doctors in assessment of the patients' condition and prognosis.

As a common marker used for evaluating the balance of fluid and electrolyte in clinical practice (4–7), plasma osmolarity can be calculated easily from serum sodium, potassium, glucose, and blood nitrogen urea (8). Previous clinical studies have shown that plasma osmolarity is associated with prognosis of many diseases, such as stroke (9), intracerebral hemorrhage (10), and gastrointestinal diseases (11). Plasma osmolarity is also tightly related to a higher rate of mortality and adverse cardiac events in patients with heart failure (12, 13). Likewise, in patients with coronary artery disease undergoing percutaneous coronary intervention (PCI), higher plasma osmolarity was shown to be associated with higher mortality and acute kidney injury (14, 15). Plasma osmolarity is also closely associated with the severity of disease, in-hospital mortality, and other adverse outcomes in critically ill patients (11). However, no research has been done to explore the influence of plasma osmolarity on the prognosis of CICU patients. Therefore, the purpose of this study was to identify the association between plasma osmolarity and in-hospital mortality in CICU patients.

## Method

### Population Selection Criteria and Definition of Plasma Osmolarity

**Figure 1:**
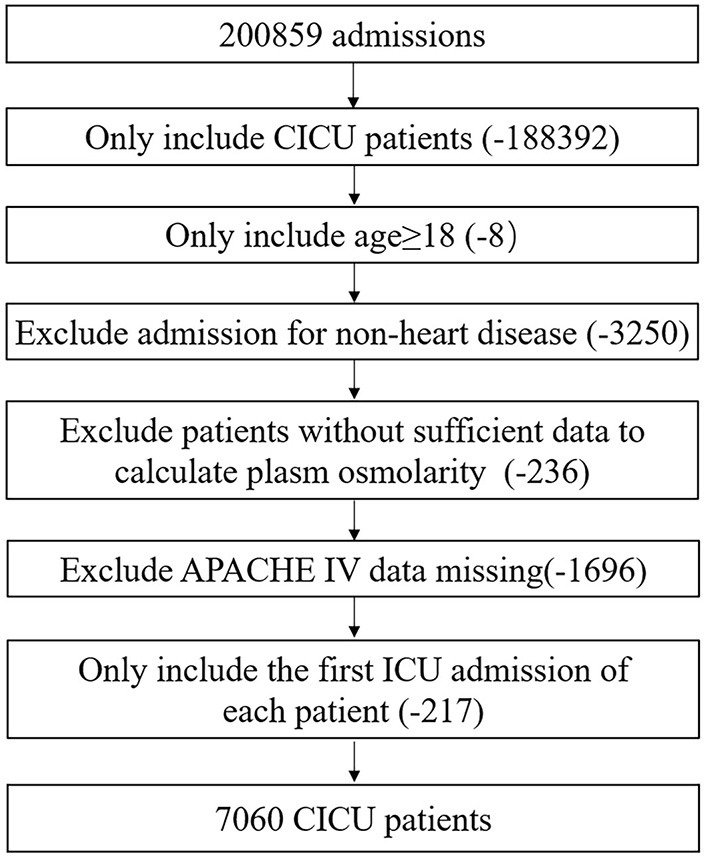
Flow chart of study population. CICU, cardiac intensive care unit; APACHE IV, acute physiology and chronic health evaluation IV.

As shown in Figure 1, all adult CICU patients at their first admission were eligible. Patients meeting the following criteria were excluded: (1) age under 18 years; (2) hospital admission for non-heart disease; (3) insufficient data to calculate plasma osmolarity; and (4) Acute Physiology and Chronic Health Evaluation IV (APACHE IV) data missing. A total of 7,060 CICU patients were included.

Plasma osmolarity was calculated as follows: 2 × [serum sodium concentration (mmol/L)] + 2 × [serum potassium concentration (mmol/L)] + [blood glucose (mmol/L)] + [blood nitrogen urea (mmol/L)] (8). Initial plasma osmolarity referred to the plasma osmolarity obtained from the first blood test after admission, while maximum osmolarity referred to the maximum plasma osmolarity during hospitalization. Plasma osmolarity was calculated from the serum sodium, potassium, glucose, and blood nitrogen urea levels measured at the same time.

### Data Extraction

The data used in this study were taken from eICU Collaborative Research Database (16), which collected information on 200,859 admissions from 208 hospitals in the United States between 2014 and 2015. This database is available at: https://doi.org/10.13026/C2WM1R, and the author was granted access to the database through Protecting Human Research Participants exam (certificate number: 9,728,458).

The following data were collected: demographics (age, gender, and race), vital signs (blood pressure, heart rate, respiration rate, oxygen saturation), body mass index, diagnoses and comorbidities [coronary artery disease, acute coronary syndrome, ST-elevation myocardial infarction (STEMI), non-ST-elevation myocardial infarction (NSTEMI), congestive heart failure, arrhythmias, cardiac arrest, atrial fibrillation, ventricular arrhythmias, atrioventricular block, cardiomyopathy, valve disease, shock, pulmonary embolism, pulmonary hypertension, hypertension, diabetes, chronic obstructive pulmonary disease (COPD), respiratory failure, chronic kidney disease, acute kidney injury, malignancy, stroke, sepsis], laboratory parameters (white blood cells, red blood cells, platelets, hemoglobin, hematocrit, glucose, creatinine, blood nitrogen urea, sodium, potassium), medication use [antiplatelet, oral anticoagulants, beta-blockers, angiotensin-converting enzyme inhibitor/angiotensin receptor blocker (ACEI/ARB), statins], acute physiology score (APS), and Acute Physiology and Chronic Health Evaluation IV (APACHE IV) (17).

### Grouping and Outcomes

**Figure 2:**
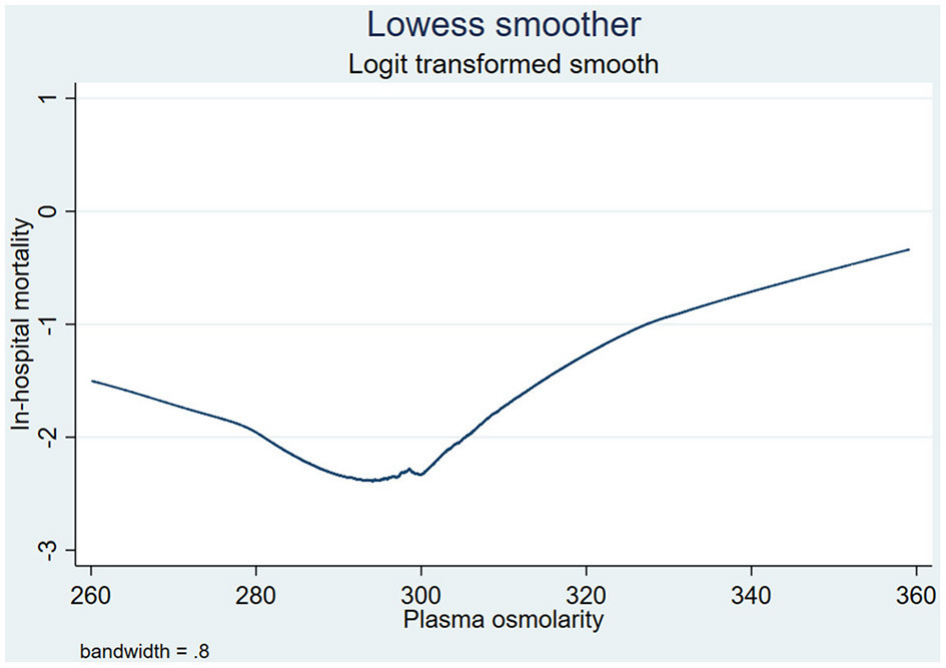
Association between plasma osmolarity and in-hospital mortality presented through Lowess smoothing. Lowess, local weighted regression.

In clinical practice, we usually consider 285–307 mmol/L as a normal range of plasma osmolarity (8); however, according to the Lowess smoothing curve (Figure 2), we found that in-hospital mortality was the lowest when plasma osmolarity ranged from 290 to 300 mmol/L. Therefore, we decided to use osmolarity of 290–300 mmol/L as the reference group in binary logistic regression analysis. In order to better explore the association between plasma osmolarity and in-hospital mortality of CICU patients, all of the enrolled patients were divided into seven groups according to their initial plasma osmolarity: group 1 (<280 mmol/L), group 2 (280–290 mmol/L), group 3 (290–300 mmol/L), group 4 (300–310 mmol/L), group 5 (310–320 mmol/L), group 6 (320–330 mmol/L), and group 7 (≥ 330 mmol/L).

The primary outcome was in-hospital mortality. Secondary outcomes were length of CICU stay and length of hospital stay.

### Statistical Analysis

Normally distributed continuous variables were expressed as mean ± standard deviation (SD) and compared between the groups using Student's *t*-test. Skewed data were expressed as median and interquartile range (IQR) and were compared using the Kruskal–Wallis test or the Mann–Whitney *U*-test. Categorical variables were expressed as a number (percentage) and compared between the groups using the chi-square test.

The relationship between plasma osmolarity and in-hospital mortality was identified by binary logistic regression analysis, and the results were expressed as odds ratio (OR) with 95% confidence interval (CI). Covariates were selected on basis of statistical analysis and clinical suspicion that the factors may modulate the result. The curve in line with overall trend was drawn by local weighted regression (Lowess). All the tests were two-sided, and P <0.05 was considered statistically significant. All of the data analyses were performed in Stata V.15.1.

## Results

### Subjects and Baseline Characteristics

As shown in Figure 1, a total of 7,060 CICU patients were enrolled after screening step by step; most of them were White and male. Baseline characteristics of survivors and non-survivors are shown in Table 1. Initial plasma osmolarity and maximum plasma osmolarity of all the patients were 302.2 ± 14.4 and 308.4 ± 15.6 mmol/L, respectively. Non-survivors had higher initial plasma osmolarity (308.1 ± 18.1 vs. 301.4 ± 13.7, *P* < 0.001) and maximum plasma osmolarity (321.0 ± 19.9 vs. 306.7 ± 14.1, *P* < 0.001) than survivors. Non-survivors were more likely to have lower blood pressure, oxygen saturation, and body mass index, but higher heart rate and respiration rate. Moreover, non-survivors more often presented congestive heart failure, cardiac arrest, atrial fibrillation, ventricular arrhythmias, shock, COPD, respiratory failure, chronic kidney disease, acute kidney injury, malignancy, stroke, and sepsis, but less commonly coronary artery disease, acute coronary syndrome, STEMI, and hypertension. Non-survivors also had higher white blood cell count, glucose, creatinine, blood nitrogen urea, sodium, and potassium levels, but lower red blood cell and platelet counts, hemoglobin, and hematocrit. Non-survivors less often received oral anticoagulant, antiplatelet, beta-blocker, ACEI/ARB, and statin therapy. APS and APACHE IV of non-survivors were significantly higher than those of survivors.

**Table 1:** Baseline characteristics between survivors and non-survivors.

**Characteristics**	**Total (*n* = 7,060)**	**Survivors (*n* = 6,207)**	**Non-survivors (*n* = 853)**	***P*-value**
Age (years)	65.6 ± 15.2	65.1 ± 15.3	69.4 ± 13.7	<0.001
Gender, *n* (%)				0.701
Male	3,958 (56.1)	3,485 (56.2)	473 (55.5)	
Female	3,102 (43.9)	2,722 (43.9)	380 (44.6)	
Ethnicity, *n* (%)				<0.001
Caucasian	4,989 (70.7)	4,366 (70.3)	623 (73.0)	
African American	1,185 (16.8)	1,022 (16.5)	163 (19.1)	
Other	886 (12.6)	819 (13.2)	67 (7.9)	
**Vital signs**				
Systolic blood pressure (mmHg)	122.3 ± 19.7	123.7 ± 19.3	111.7 ± 19.4	<0.001
Diastolic blood pressure (mmHg)	66.1 ± 11.3	66.7 ± 11.2	61.3 ± 10.8	<0.001
Mean blood pressure (mmHg)	82.3 ± 13.0	83.1 ± 12.9	76.0 ± 12.3	<0.001
Heart rate (beats/min)	87.4 ± 22.4	86.4 ± 21.9	95.0 ± 24.1	<0.001
Respiration rate (beats/min)	21.0 ± 6.7	20.7 ± 6.5	22.9 ± 7.7	<0.001
Oxygen saturation (%)	96.3 ± 5.3	96.5 ± 4.4	94.4 ± 9.5	<0.001
Body mass index (kg/m^2^)	29.1 ± 7.5	29.2 ± 7.4	28.4 ± 8.1	0.006
**Diagnoses and comorbidities**, ***n*** **(%)**				
Congestive heart failure	1,396 (19.8)	1,200 (19.3)	196 (23.0)	0.012
Coronary artery disease	2,619 (37.1)	2,417 (38.9)	202 (23.7)	<0.001
Acute coronary syndrome	1,646 (23.3)	1,518 (24.5)	128 (15.0)	<0.001
STEMI	688 (9.8)	641 (10.3)	47 (5.5)	<0.001
NSTEMI	499 (7.1)	441 (7.1)	58 (6.8)	0.774
Arrhythmias	2,205 (31.2)	1,935 (31.2)	270(32.7)	0.777
Cardiac arrest	577 (8.2)	270 (4.4)	307 (36.0)	<0.001
Atrial fibrillation	1,260 (17.9)	1,077 (17.4)	183 (21.5)	0.003
Ventricular arrhythmias	114 (1.6)	83 (1.3)	31 (3.6)	<0.001
Atrioventricular block	176 (2.5)	161 (2.6)	15 (1.8)	0.142
Cardiomyopathy	419 (5.9)	370 (6.0)	49 (5.7)	0.802
Valve disease	182 (2.6)	157 (2.5)	25 (2.9)	0.488
Shock	1,951 (27.6)	1,534 (24.7)	417 (48.9)	<0.001
Pulmonary embolism	143 (2.0)	122 (2.0)	21 (2.5)	0.335
Pulmonary hypertension	76 (1.1)	65 (1.1)	11 (1.3)	0.520
Hypertension	2,019 (28.6)	1,868 (30.1)	151 (17.7)	<0.001
Diabetes	1,306 (18.5)	1,146 (18.5)	160 (18.8)	0.836
COPD	717 (10.2)	610 (9.8)	107 (12.5)	0.014
Respiratory failure	1,894 (26.8)	1,416 (22.8)	478 (56.0)	<0.001
Chronic kidney disease	982 (13.9)	821 (13.2)	161 (18.9)	<0.001
Acute kidney injury	1,178 (16.7)	895 (14.4)	283 (33.2)	<0.001
Malignancy	371 (5.3)	294 (4.7)	77 (9.0)	<0.001
Stroke	262 (3.7)	212 (3.4)	50 (5.9)	<0.001
Sepsis	1,396 (19.8)	1,113 (17.9)	283 (33.2)	<0.001
**Laboratory parameters**				
White blood cell (10^9^/L)	11.7 ± 8.4	11.3 ± 7.9	14.6 ± 11.1	<0.001
Red blood cell (10^9^/L)	4.1 ± 0.8	4.1 ± 0.8	3.9 ± 0.8	<0.001
Platelet (10^9^/L)	226.6	227.8 ± 96.4	217.4 ± 108.3	0.004
Hemoglobin (g/dL)	12.1 ± 2.5	12.2 ± 2.5	11.5 ± 2.5	<0.001
Hematocrit (%)	36.7 ± 7.0	36.9 ± 7.0	35.4 ± 7.4	<0.001
Glucose (mmol/L)	8.9 ± 5.1	8.7 ± 5.0	10.2 ± 6.0	<0.001
Creatinine (mg/dL)	1.69 ± 1.48	1.64 ± 1.48	2.02 ± 1.47	<0.001
Blood nitrogen urea (mmol/L)	28.7 ± 21.7	27.4 ± 20.6	38.1 ± 26.3	<0.001
Sodium (mmol/L)	137.2 ± 5.3	137.2 ± 5.2	137.6 ± 6.4	0.043
Potassium (mmol/L)	4.2 ± 0.8	4.2 ± 0.7	4.4 ± 0.9	<0.001
**Medication use**, ***n*** **(%)**				
Antiplatelet	3,396 (48.1)	3,078 (49.6)	318 (37.3)	<0.001
Oral anticoagulants	767 (10.9)	710 (11.4)	57 (6.7)	<0.001
Beta-blockers	3,034 (43.0)	2,795 (45.0)	239 (28.0)	<0.001
ACEI/ARB	1,914 (27.1)	1,805 (29.1)	109 (12.8)	<0.001
Statin	2,150 (30.5)	1,999 (32.2)	151(17.7)	<0.001
APS	38 (27–55)	36 (25–49)	76 (52–106)	<0.001
APACHE IV	52 (38–70)	49 (36–64)	92 (67–121)	<0.001
Initial osmolarity (mmol/L)	302.2 ± 14.4	301.4 ± 13.7	308.1 ± 18.1	<0.001
Maximum osmolarity (mmol/L)	308.4 ± 15.6	306.7 ± 14.1	321.0 ± 19.9	<0.001

*Normally distributed continuous variables were presented as mean ± SD or median (IQR). Categorical variables were presented as number (percentage). STEMI, ST-elevation myocardial infarction; NSTEMI, non-ST-elevation myocardial infarction; COPD, chronic obstructive pulmonary disease; ACEI, angiotensin-converting enzyme inhibitor; ARB, angiotensin receptor blocker; APS, acute physiology score; APACHE IV, acute physiology and chronic health evaluation IV*.

### Association Between Osmolarity and Outcomes

The primary outcome was in-hospital mortality. Through the Lowess smoothing curve shown in Figure 2, a “U”-shaped relationship between in-hospital mortality and plasma osmolarity was found. When plasma osmolarity ranged from 290 to 300 mmol/L, in-hospital mortality of CICU patients was the lowest. Therefore, we decided to use osmolarity of 290–300 mmol/L as the reference group in binary logistic regression analysis.

Table 2 shows crude outcomes by plasma osmolarity categories. The lowest in-hospital mortality (7.2%) was observed in the group with 290–300 mmol/L osmolarity. When plasma osmolarity was >290 mmol/L, as plasma osmolarity increased, in-hospital mortality increased significantly (≥330 vs. 290–300 mmol/L: 31.6 vs. 7.2%, respectively). When plasma osmolarity was below 300 mmol/L, as plasma osmolarity decreased, in-hospital mortality increased significantly (<280 vs. 290–300 mmol/L: 13.0 vs. 7.2%, respectively). Higher in-hospital mortality was confirmed in both lower and higher plasma osmolarity group, which was similar with the conclusion drawn by Lowess smoothing shown in Figure 2. Moreover, the lengths of CICU and hospital stays were the lowest in the 290–300 mmol/L group; in contrast, the lengths of CICU and hospital stays were prolonged in both hyposmolarity and hyperosmolarity groups (Table 2).

**Table 2:** Outcomes by osmolarity categories in CICU patients.

**Outcome**	**Osmolarity (mmol/L)**							
	** <280**	**280–290**	**290–300**	**300–310**	**310–320**	**320–330**	**≥330**	***P*-value**
	**(*n* = 231)**	**(*n* = 732)**	**(*n* = 2,283)**	**(*n* = 2290)**	**(*n* = 917)**	**(*n* = 363)**	**(*n* = 244)**	
In-hospital mortality, *n* (%)	30 (13.0)	71 (9.7)	165 (7.2)	243 (10.6)	173 (18.9)	94 (25.9)	77 (31.6)	<0.001
Length of CICU stay (days)	2.2 (1.4–4.6)	2.0 (1.1–3.9)	1.8 (1.0–3.1)	1.9 (1.1–3.4)	2.2 (1.2–4.1)	2.7 (1.5–5.0)	3.3 (1.6–6.0)	<0.001
Length of hospital stay (days)	5.7 (3.0–10.7)	5.2 (2.9–9.9)	4.6 (2.5–8.9)	5.0 (2.8–9.2)	5.9 (3.1–10.3)	7.4 (3.6–12.2)	7.9 (4.3–14.9)	<0.001

*Lengths of CICU and hospital stays were skewed. Therefore, they were presented as median (IQR). Categorical variables were presented as number (percentage). CICU, cardiac intensive care unit*.

**Figure 3:**
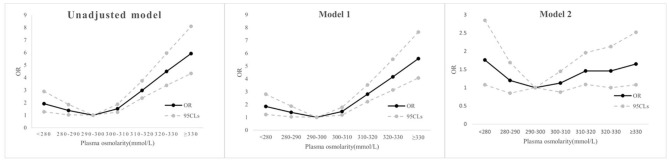
Line graphs reflecting the trend of change in OR of in-hospital mortality in unadjusted model, model 1, and model 2. OR, odds ratio; CI, confidence interval.

As shown in Table 3, in unadjusted logistic regression model, with the 290–300 mmol/L group serving as the reference group, both hyposmolarity (<280 vs. 290–300 mmol/L: OR, 95% CI: 1.92, 1.27–2.90, *P* = 0.002) and hyperosmolarity (≥330 mmol/L vs. 290–300 mmol/L: OR, 95% CI: 5.92, 4.33–8.09, *P* < 0.001) were related to the increased risk of in-hospital mortality. When plasma osmolarity was >290 mmol/L, the risk of in-hospital mortality increased gradually as plasma osmolarity increased. When plasma osmolarity was below 300 mmol/L, the risk of in-hospital mortality increased gradually as plasma osmolarity decreased. After adjusting for age, gender, and ethnicity in the model 1, the conclusion was basically consistent with that of the unadjusted model. After adjusting for more possible confounding variables in the model 2, the association between osmolarity and in-hospital mortality was attenuated but still remained statistically significant. Both hyposmolarity (<280 vs. 290–300 mmol/L: OR, 95% CI: 1.76, 1.08–2.85, *P* = 0.023) and hyperosmolarity (≥330 mmol/L vs. 290–300 mmol/L: OR, 95% CI: 1.65, 1.08–2.52, *P* = 0.021) were independently associated with the increased risk of in-hospital mortality. OR values increased gradually as plasma osmolarity increased when plasma osmolarity was >290 mmol/L; when plasma osmolarity was below 300 mmol/L, OR values increased gradually as plasma osmolarity decreased. Figure 3 vividly presents the change of OR with the change of osmolarity groups in the unadjusted model, model 1, and model 2.

**Table 3:** The association between in-hospital mortality and osmolarity (mmol/L).

	**Unadjusted**		**Model 1**		**Model 2**	
	**OR(95% CIs)**	***P*-value**	**OR(95% CIs)**	***P*-value**	**OR(95% CIs)**	***P*-value**
Osmolarity (<280)	1.92 (1.27–2.90)	0.002	1.85 (1.22–2.80)	0.004	1.76 (1.08–2.85)	0.023
Osmolarity (280–290)	1.38 (1.03–1.85)	0.031	1.39 (1.04–1.87)	0.027	1.20 (0.85–1.69)	0.289
Osmolarity (290–300)	1.0 (reference)		1.0 (reference)		1.0 (reference)	
Osmolarity (300–310)	1.52 (1.24–1.87)	<0.001	1.45 (1.18–1.78)	<0.001	1.13(0.88–1.45)	0.351
Osmolarity (310–320)	2.98 (2.37–3.75)	<0.001	2.80 (2.22–3.53)	<0.001	1.46 (1.09–1.96)	0.012
Osmolarity (320–330)	4.49 (3.38–5.95)	<0.001	4.16 (3.13–5.53)	<0.001	1.46 (1.00–2.13)	0.052
Osmolarity (≥330)	5.92 (4.33–8.09)	<0.001	5.58 (4.07–7.65)	<0.001	1.65 (1.08–2.52)	0.021

*Models were derived from binary logistic regression analysis. Unadjusted model: unadjusted. Model 1: adjusted for age, gender, ethnicity. Model 2: adjusted for age, gender, ethnicity, systolic blood pressure, diastolic blood pressure, mean blood pressure, heart rate, respiration rate, congestive heart failure, coronary artery disease, acute coronary syndrome, STEMI, NSTEMI, cardiac arrest, ventricular arrhythmias, shock, hypertension, diabetes, respiratory failure, acute kidney injury, sepsis, stroke, malignancy, white blood cell, red blood cell, hemoglobin, creatinine, ACEI/ARB, beta-blockers, statin, and oral anticoagulants, APS, APACHE IV. OR, odds ratio; CI, confidence interval*.

## Discussion

This study identified the association between plasma osmolarity and in-hospital mortality in CICU patients. A “U”-shaped relationship between plasma osmolarity and in-hospital mortality was observed. With the group of 290–300 mmol/L serving as the reference group, both hyposmolarity and hyperosmolarity were associated with the increased risk of in-hospital mortality, even after adjusting for possible confounding variables. The lengths of CICU and hospital stays were prolonged in both hyposmolarity and hyperosmolarity groups.

As a common clinical marker to evaluate the balance of fluid and electrolytes (4–7), plasma osmolarity can be easily calculated from the concentrations of serum sodium, potassium, glucose, and blood nitrogen urea (8). Plasma osmolarity is the most commonly used indicator of hydration (18), which can influence cell size and function (19). Therefore, changes in plasma osmolarity can reflect changes in cell function. A great number of studies have been done on plasma osmolarity, and there is sufficient evidence that plasma osmolarity is associated with the prognosis of many diseases, such as stroke (9), intracerebral hemorrhage (10), and acute pulmonary embolism (20). Moreover, recent studies have shown a correlation between plasma osmolarity and cardiovascular diseases. A single-center retrospective study with 1,927 patients after PCI showed that the rate of acute kidney injury and 1-year mortality increased significantly as plasma osmolarity increased (14). Another study, which enrolled 985 patients with acute coronary syndrome undergoing PCI, confirmed higher mortality in the higher osmolarity group (15). In patients with STEMI, higher rates of all-cause mortality, recurrent myocardial infarction, and revascularization were found in those with higher plasma osmolarity (21). Previous studies also showed that both low and high plasma osmolarity were related to more cardiovascular deaths, deterioration of cardiac function, and rehospitalization in patients with heart failure (12, 13). In this study exploring the relationship between plasma osmolarity and in-hospital mortality in CICU patients, we came to a similar conclusion that plasma osmolarity was closely associated with in-hospital mortality. Moreover, through Lowess smoothing, we found a “U”-relationship between in-hospital mortality and osmolarity, which provided a more graphic description of the overall trend.

Plasma osmolarity is mainly determined by serum sodium, chloride, potassium, blood glucose, and blood nitrogen urea. Hypernatremia, hyperchloremia, hyperkalemia, hyperglycemia, and high urea contribute to high plasma osmolarity. Hypernatremia was shown to be associated with higher mortality and more cardiovascular diseases in older men (22). Another study confirmed that increased hypernatremia was associated with higher perioperative 30-day mortality (23). For patients with intracranial hemorrhage, hypernatremia was associated with more adverse cardiac events (24). Patel et al. found that hyperchloremia was independently associated with acute kidney injury in patients with STEMI undergoing PCI (25). Hyperkalemia can lead to malignant arrhythmia and increase mortality (26). Hyperglycemia is very common in clinical practice and it is related to higher mortality and more adverse cardiac events in patients with or without diabetes (27). A prospective study with 1,667 patients diagnosed with acute coronary syndrome showed that high blood nitrogen was associated with more adverse cardiac events and higher mortality (28). These studies can explain why high plasma osmolarity leads to high mortality, which can also explain the results of our study. The lengths of CICU and hospital stays were prolonged in both the hyposmolarity and the hyperosmolarity groups, indicating that patients with hyposmolarity or hyperosmolarity had a more complex condition and therefore required a longer treatment. The increased lengths of CICU and hospital stays imposed not only the financial but also mental burden on patients. In exceptional cases, some patients may abandon treatment because of financial problems. Therefore, more attention to plasma osmolarity of CICU patients is needed.

Changes in plasma osmolarity can provide guidance for clinical practice. Usually, the clinicians tend to pay more attention to the outliers, but when all the variables are within the normal range but close to the upper limit of the normal value, plasma osmolarity will increase significantly. At this time, plasma osmolarity can better reflect the patient's condition and give the clinician a hit. The independent association between in-hospital mortality and plasma osmolarity was confirmed in this study. As a readily accessible and inexpensive prognostic marker, plasma osmolarity is clinically valuable for critically ill patients admitted to CICU, especially in some cases that more complex prognostic score can't be calculated, for example, the patient is unable to undergo complex examination or the patient is in a remote area without the means to do so, plasma osmolarity may alert the clinicians.

We confirmed the association between plasma osmolarity and in-hospital mortality in CICU patients in this study, which is convenient for clinical use. The multicenter and large sample size makes the conclusion more reliable. However, some limitations in this study should be noted. First, bias was inevitable due to the retrospective nature of the study. Second, some important information, such as left ventricular ejection fraction and information about smoking and alcohol, could not be collected. In general, the variables included in the model determine the accuracy of the model; thus, the accuracy of the model was likely affected by the missing variables. Third, we were not able to dynamically observe plasma osmolarity. Fourth, although the optimal equation was used, the calculated plasma osmolarity cannot be the exactly the same as the real plasma osmolarity.

## Conclusion

A “U”-shaped relationship between plasma osmolarity and in-hospital mortality was observed. The lowest in-hospital mortality was shown in the group with 290–300 mmol/L osmolarity; patients with hyposmolarity or hyperosmolarity had higher in-hospital mortality. With the group with 290–300 mmol/L osmolarity serving as the reference group, both hyposmolarity and hyperosmolarity were shown to be independently associated with the increased risk of in-hospital mortality.

## Data Availability Statement

The original contributions presented in the study are included in the article/Supplementary Materials, further inquiries can be directed to the corresponding author/s.

## Ethics Statement

Ethical review and approval was not required for the study on human participants in accordance with the local legislation and institutional requirements. The ethics committee waived the requirement of written informed consent for participation.

## Author Contributions

GZ and YZ contributed to study design, data analysis, and article writing. JW and YL contributed to data collection. All authors contributed to the article and approved the submitted version.

## Conflict of Interest

The authors declare that the research was conducted in the absence of any commercial or financial relationships that could be construed as a potential conflict of interest.

## Supplementary Material

Results of covariates in multiple logistic regression analysis (Model 2) were presented in the Supplementary Materials.
